# Widening access to penicillin allergy assessment in the United Kingdom—a proposed implementation plan for the National Health Service (NHS)

**DOI:** 10.1093/jacamr/dlaf240

**Published:** 2026-01-07

**Authors:** Catherine E Porter, Caity Roleston, Claire Bethune, Jenny Boards, Colin S Brown, Ian Clarke, Joanne Fielding, Philip Howard, Conor Jamieson, Siraj A Misbah, Andrew C Moss, Sue H Pavitt, Neil Powell, Louise Savic, Sinisa Savic, Mamidipudi Thirumala Krishna, Sarah Tonkin-Crine, Iestyn Williams, Jonathan A.T. Sandoe

**Affiliations:** Dental Translational and Clinical Research Unit, School of Dentistry, University of Leeds, Leeds, UK; Nuffield Department of Primary Care Health Sciences, University of Oxford, Oxford, UK; Peninsula Immunology and Allergy Service, University Hospitals Plymouth NHS Trust, Plymouth, UK; PPIE Contributor, Dental Translational and Clinical Research Unit, part of the NIHR Leeds Clinical Research Facility, School of Dentistry, University of Leeds, Leeds, UK; Antimicrobial Resistance & Healthcare-Associated Infections Division, UK Health Security Agency, London, UK; NIHR Health Protection Research Unit in Healthcare Associated Infections and Antimicrobial Resistance (NIHR200915), University of Oxford, Oxford, UK; PPIE Contributor; Dental Translational and Clinical Research Unit, School of Dentistry, University of Leeds, Leeds, UK; Regional Antimicrobial Stewardship Lead, NHS England, North-East and Yorkshire, Newcastle, UK; School of Healthcare, University of Leeds, Leeds, UK; Medical Directorate, NHS England (Midlands), Birmingham, UK; Department of Clinical Immunology, Oxford University Hospitals NHS Foundation Trust, Oxford, UK; Egton Medical Information Systems Limited (t/a Optum), Fulford Grange, Micklefield Lane, Rawdon, Leeds, UK; Dental Translational and Clinical Research Unit, School of Dentistry, University of Leeds, Leeds, UK; Pharmacy Department, Royal Cornwall Hospitals NHS Trust, Truro, Cornwall, UK; Department of Anaesthesia, Leeds Teaching Hospitals NHS Trust, Leeds, UK; Leeds Institute of Rheumatic and Musculoskeletal Medicine, University of Leeds, Leeds, UK; Department of Clinical Immunology and Allergy, St James’s University Hospital, Leeds Teaching Hospitals NHS Trust, Leeds, UK; Department of Immunology and Immunotherapy, School of Infection, Inflammation and Immunology, University of Birmingham, Birmingham, UK; Department of Allergy and Immunology, University Hospitals Birmingham NHS Foundation Trust, Birmingham, UK; Nuffield Department of Primary Care Health Sciences, University of Oxford, Oxford, UK; NIHR Health Protection Research Unit in Healthcare Associated Infections and Antimicrobial Resistance (NIHR200915), University of Oxford, Oxford, UK; Health Services Management Centre, University of Birmingham, Edgbaston, Birmingham, UK; Healthcare Associated Infection Group, Leeds Institute of Medical Research, School of Medicine, University of Leeds, Leeds, UK; Department of Medical Microbiology, Leeds Teaching Hospitals NHS Trust, Leeds, UK

## Abstract

Globally, there is increasing evidence that incorrect penicillin allergy labels negatively affect patient outcomes, antibiotic prescribing and antimicrobial resistance, leading to growing concern about this patient safety issue and how to resolve it. While many millions of patients worldwide have incorrect penicillin allergy labels, there are too few specialist allergists and a lack of ‘point-of-care’ tests to address this problem. Numerous research studies now provide evidence of the feasibility and importance of widening access to penicillin allergy assessment. Researchers from two UK-based studies (SPACE and ALABAMA), in collaboration with key stakeholders including patient representatives, gave their views to shape a high-level implementation plan to facilitate widening access to penicillin allergy assessment in the UK. This Viewpoint describes the basis of the implementation plan and summarizes the key actions required for successful delivery. While the plan is intended for the UK, we hope to promote international shared learning and collaboration to address this global problem informed by the UK context.

## Aims

This Viewpoint aims to set out what we feel needs to happen to enable wider access to penicillin allergy assessment for patients within the National Health Service (NHS). We feel it is important to have assigned responsibilities and to describe where in the health system we think these responsibilities should lie. We aim to communicate a carefully considered, objective and transparent process involving multiple stakeholders, all of whom are committed to addressing this important patient safety issue. The proposed implementation plan is aimed at service delivery by both allergy specialists and non-allergy specialists as we are looking at widening access to penicillin allergy assessment for all patients.

## The problem of incorrect penicillin allergy labels in health records

The presence of an incorrect penicillin allergy label in a patient’s medical records is a driver of unnecessary over-use of non-penicillin antibiotics and is associated with treatment failures and more antimicrobial resistance.^1–4^ Ninety percent of patients with a penicillin allergy label are not allergic when formally assessed.^4^ In England alone there are likely to be >3.5 million adults incorrectly labelled as allergic to penicillin. Incorrect penicillin allergy labels often arise because symptoms of infection or non-immune adverse reactions are misdiagnosed as allergic reactions. Penicillin allergy labels are also associated with worse health outcomes, such as increased mortality and admission to hospital and intensive care.^5^ It is not known why patients with a penicillin allergy label experience worse health outcomes but it is likely to be driven by sub-optimal efficacy, side effects and other complications of the alternative antibiotics they receive.^1,2^

Of adults registered with a general practice in England, 6%–8% have a penicillin allergy label in their health record but prevalence in the general population varies by country, for example 2% and 13% in the Netherlands and USA, respectively.^1,5–7^ The prevalence rises to 15%–20% in patients in secondary care.^8^ About 5% of children in the USA have a penicillin allergy label, but specific data for children from the UK are lacking.^4,9^ Incorrect penicillin allergy labels are considered a global problem and the international importance of penicillin allergy assessment is emphasized in WHO antimicrobial stewardship guidance.^10^ For the UK, the national antimicrobial resistance 5-year national action plan (NAP) for 2024–2029 specifically highlights the importance of access to penicillin allergy de-labelling services and increasing use of UK-AWaRe ‘Access’ (UK Access, Watch and Reserve, and Other antibiotic classification list) antimicrobials, which penicillin allergy de-labelling will contribute towards.^11,12^ A core target (Target 4b) of the UK NAP is to achieve 70% of total use of antibiotics from the Access category across the human healthcare system by 2029.

## Current provision of penicillin allergy assessment and Allergy Specialist input in the NHS

In this article, penicillin allergy assessment refers to the process of taking an allergy history and general medical history from the patient, assessing their risk of true allergy, testing according to this risk assessment and updating medical records accordingly. Penicillin allergy testing involves either being given a test dose of penicillin (oral challenge test, OCT) directly or having skin testing before an OCT. Some patients who are at very low risk of a true allergy can have their penicillin allergy label removed directly, without testing.^13^

National Institute of Health and Care Excellence (NICE) guidelines on management of drug allergy provide guidance on which patients are eligible for penicillin allergy assessment in the NHS.^14^ This NICE guideline currently limits penicillin allergy assessment to the subset of patients with a probably frequent need for penicillins and those who can only be treated with a penicillin.^14^ The British Society for Allergy and Clinical Immunology (BSACI) has acknowledged the need to address the problem of incorrect penicillin allergy labels and the need to expand the workforce capable of delivery, and has published guidelines for non-specialist de-labelling to facilitate this.^13^ The BSACI guideline requires allergy or immunology ‘oversight’ of penicillin allergy assessment but does not define what this means. Systematic review evidence has confirmed that a range of non-specialists, including pharmacists, doctors, nurses, nurse practitioners, physician associates, medical students and pharmacy students, can safely de-label patients at low risk of serious allergic reactions.^15^ Specialist assessment of penicillin allergy has traditionally been delivered by allergy specialists but there are too few of these to deliver penicillin allergy assessment at the scale required, a problem that is seen across most health systems.^16,17^ Pragmatically, one solution is for the allergy assessment workforce to be expanded using non-specialists to de-label patients with a low risk of a true penicillin allergy.

## UK research supporting the evidence base for removing allergy labels

There is growing evidence of the feasibility and importance of widening access to penicillin allergy assessment within the UK health infrastructure. The Spurious Penicillin Allergy in Secondary Care (SPACE)^18^; Penicillin Allergy, Antibiotic Resistance and Patient health OuTcomes (PARROT)^19^; Penicillin allergy status and its effect on antimicrobial prescribing, patient outcomes, and antimicrobial resistance (ALABAMA)^20^ and Removing Erroneous Penicillin Allergy Labels (REPeAL)^21^ studies are all National Institute for Health and Care Research (NIHR) funded research projects looking at the problem of incorrect penicillin allergy labels. The SPACE and REPeAL studies have provided evidence that it is feasible for non-specialists to safely deliver penicillin allergy assessments to low-risk patients in secondary care in the UK NHS and this is supported by both patients and healthcare workers.^15,22–25^ In a randomized controlled trial delivered under the close supervision of specialist allergists, the ALABAMA study has provided the first randomized evidence of the therapeutic benefits of penicillin allergy de-labelling in primary care patients.^26^

## Widening access to penicillin allergy assessment (PAA) in the NHS

### Developing our approach

The SPACE and ALABAMA research teams jointly hosted an event with a wide range of invited stakeholders from across the health system in London in September 2024, to plan how to facilitate widening access to penicillin allergy assessment (PAA) in the NHS. The event comprised a morning of dissemination presentations from both studies and a patient contributor. The afternoon was dedicated to discussing ideas for widening access to PAA and relevant policy considerations needed to support this. Refer to Supplemental Materials Appendix X (available as Supplementary data at *JAC-AMR* Online) for further details.

During the afternoon discussion, attendees were asked to share their views on what would represent successful wider provision of de-labelling for penicillin allergy in the UK in 2 years’ time and how this might be achieved. Members of the ALABAMA research team subsequently collated and undertook thematic analysis of the attendees’ written responses.^27^ They produced a summary document of the themes (see Supplemental Materials Appendix X). Two meetings were then held between the research teams to develop and refine an agreed action list (Table 1) derived from these themes, forming the proposed implementation plan. Recommendations from the stakeholder event have not been formally commissioned.

**Table 1.** Key actions for implementation. The key actions for implementation identified have been categorized into nine broad themes which are outlined below

Key actions for implementation
(i) Translating aspiration into action
Establish a Penicillin Allergy Network and working groups Submit advisory paper to the Department of Health and Social Care Antimicrobial Prescribing, Resistance and Healthcare Associated Infection Group Work with the National Allergy Strategy Group to include penicillin allergy in strategic planning
(ii) National level support/endorsement
Request update of NICE guideline CG183^14^ ‘Drug allergy: Diagnosis and management of drug allergy in adults, children and young people’ penicillin allergy components Request development of standardized toolkits/resources for non-specialist PAA Obtain endorsement for non-specialist delivered PAA from senior medical authorities across devolved nations Collaborate with existing stakeholder groups (e.g. National Allergy Strategy Group, allergy working group and British Society for Allergy and Clinical Immunology, and British Society for Immunology-Clinical immunology professional network, British Society for Antimicrobial Chemotherapy and British Infection Association)
(iii) Electronic health record(s):
Develop information technology policy/strategy document for penicillin allergy Work with providers to update EHR systems to facilitate documentation of PAA and align with NICE guidelines Develop electronic health record-supported patient identification processes Standardized set of SNOMED codes for PAA, testing and de-labelling to be added to UK directory
(iv) Education and training:
Develop and deliver standardized PAA training model and resources for non-allergy specialist healthcare professionals Identify exemplar case studies of local non-specialist delivery of PAA
(v) Workforce and resources:
Ensure all primary care patient records have up-to-date drug allergy histories in line with NICE CG183 standards Identify local penicillin allergy de-labelling champions Establish a record of PAA services provided by early adopters in the NHS Provide local facilities for PAA and de-labelling
(vi) Research needs:
Identify research gaps and communicate to NIHR for consideration for a ‘themed funding call’ Specific research/implementation gaps identified: – which patients or patient groups would benefit most from PAA? – can patients be risk stratified according to their EHR data? – which patients require specialist allergy- immunology input? – what is the burden of penicillin allergy in different ethnic minority/socio-economic groups, development of culturally tailored approaches to enhance patient and community engagement? – optimal drug provocation testing dose and duration?
(vii) Communication plan:
Public information campaign to raise awareness of the existence of incorrect penicillin allergy labels and their consequences, and what can be done about it. Designed and delivered to minimize unintended consequences. Communication packs for healthcare professionals and commissioners (Integrated Care Boards) for dissemination to GPs.
(viii) Local implementation/delivery:
Deliver implementation of requirement for up-to-date drug allergy histories in line with NICE CG183 as part of Medicines Optimization processes (as part of prescribing incentive scheme examples for Integrated Care Boards) Update National Medicines Reconciliation guidance for hospitals, to include updating antibiotic allergy histories Develop local communication plan (e.g. present updates at Integrated Care Board’s Pharmacy Leaders webinars) Develop and implement a robust PAA patient pathway Describe existing models of delivery by early adopters
(ix) Measuring success (dependent on SNOMED codes)
Mechanisms for monitoring performance PERFORMANCE MEASURES: Number of patients assessed. Number of patients de-labelled. Adverse events associated with assessment recorded performance and safety established. Characteristics of people assessed and de-labelled (incl. ethnicity and co-morbidities). Risk categorization of patients assessed and de-labelled documented. Patient acceptability assessed.

This Viewpoint summarizes our processes and findings. We argue it is useful to have an implementation plan in the public domain and to share the process so that it is easily accessible to both a national and an international audience. Our implementation plan was developed prior to announcement of the abolition of NHS England, whose duties are to be subsumed into the Department of Health and Social Care. Further details on the planning and organization of the event in London can be found in the Supplemental Materials (Appendix X).

### Overarching principles

The agreed overarching principles for implementation were derived from attendee input at the London stakeholder event plus the subsequent meetings between the two research teams. These largely aligned with previous work:^25^

- standardize practice wherever possible
- prioritize patient safety
- ensure robust governance frameworks are in place
- embed patient and public involvement/engagement
- develop effective and consistent communication practices.

The need for patient safety as a priority is self-evident. There was strong and widespread feeling that standardized practice was vital to ensure consistent, equitable and efficient practice; colleagues wanted to be able to use centrally produced, validated resources so efforts were not duplicated. There was a strong push for practice to be standardized based on current available evidence. BSACI guidelines already state the need for robust governance frameworks and this was echoed by stakeholders.^13^ There was clear recognition that patient and public involvement/engagement was fundamental to success and required at all stages. Effective communication was discussed at all stages of the process but most discussion centred around the need for effective communication of penicillin allergy status and testing results in medical records across different healthcare providers.

### Funding

The need for funding for any additional services was a common theme during discussions, but it was considered that any changes to services would most probably need to be delivered within existing budgets. Since the London stakeholder event, the ALABAMA randomized controlled trial results have been published that showed that the PAA pathway trial intervention had an incremental cost effectiveness ratio of £10 938 per quality adjusted life year gained relative to usual clinical care and a 58% probability of being cost-effective at the £20 000 willingness-to-pay threshold.^26^ Subgroup analyses undertaken in the ALABAMA trial health economic evaluation found PAA had the greatest likelihood of being cost-effective in female patients (86%), those with two or more antibiotic prescriptions in the previous 24 months (68%), two or more NHS quality and outcomes framework (QOF) conditions (64%) and aged 65 and older (61%).^26^

### Implementation plan—key actions

The key actions for implementation identified have been categorized into nine broad themes outlined in Table 1.

Stakeholders made it clear that there needs to be a mechanism for driving change and pushing any proposed actions forward. There was wide agreement that a Penicillin Allergy Network of invested professionals and local champions would be required to bring about change, support implementation and to build resilience and sustainability into the proposal. We propose that the Penicillin Allergy Network is coordinated by the AMR Regional Antimicrobial Stewardship and Pharmacy Integration Project Manager at NHS England: North-East and Yorkshire. Our proposed implementation plan fits with the latest AMR NAP by supporting greater use of ‘Access’ category antibiotics.

To move PAA up the Department of Health and Social Care agenda, it was concluded that an implementation plan needed to be submitted to the Department of Health and Social Care’s advisory body, the Antimicrobial Prescribing, Resistance and Healthcare Associated Infection Group. A National Patient Penicillin Allergy Strategy will be needed to ensure there are coordinated and centrally led processes and commissioning pathways for both high- and low-risk patient penicillin allergy. Requirements to support non-specialist de-labelling will need to be clearly defined within the Specialised Allergy service specification.

It was acknowledged that there was some UK national governmental support for PAA in the antimicrobial resistance NAP^12^ but that this needed to be strengthened by updated/enhanced clinical guidance. In the UK, responsibility for development of national clinical guidance sits with NICE. More explicit endorsement of guidelines provided by BSACI, which were not perceived as endorsed by NICE, was also highlighted as important.^13^

It was considered essential to work with providers of GP electronic health record (EHR) systems, such as The Phoenix Partnership (TPP SystmOne) and Optum (EMIS Web) in England, to develop functionality to support PAA.

The need for standardized, centrally provided, penicillin allergy educational resources and training models for non-allergy specialist healthcare professionals, was widely expressed to ensure consistent delivery of care and efficient delivery of training. Separate to desires for funding was the need for available staff and testing facilities.

Several gaps in the current evidence base were identified and the ongoing need for high quality research, emphasized through NIHR ‘themed funding calls’.

To support local implementation, it was felt that patient assessment pathways, building on existing models of delivery by early adopters were needed. Again, with the underlying message that implementation needed to be made as easy and efficient as possible.

It was widely felt that communication campaigns to raise awareness with service commissioners, frontline healthcare professionals and the general public were all needed. While resources including a patient prioritization tool would need to be developed to avoid unintended consequences; such as large numbers of people with penicillin allergy labels asking their GP for testing before a mechanism for delivering wider access has been established.

### Summary and next steps

Considerable progress has been made defining the problem of incorrect penicillin allergy labels and establishing that they can be reversed. Among many healthcare workers there is a will to address this important patient safety issue and momentum is growing. To harness this energy there needs to be leadership by NHS England and the appointment of a project manager to organize and coordinate implementation to reduce this health inequality. The purpose of this paper is to define what this might look like.

The Penicillin Allergy Network will be organized into working groups according to members’ areas of expertise and interest, each with a focus on driving forward discrete areas for action. Those volunteering to participate in these groups will work with stakeholders within the health system to facilitate and influence widening access to PAA within the NHS.

## Supplementary Material

dlaf240_Supplementary_Data

## Transparency declarations

C.B. holds the role of National Specialty Advisor for Specialised Immunology and Allergy (NHS England funded role). C.S.B. oversees antimicrobial resistance surveillance for UK Health Security Agency as part of his role. He sits on the Global IPC Network through his role as Director of a WHO Collaborating Centre on antimicrobial resistance and healthcare-associated infection. A.C.M. was a member of the TechUK Life Science Working Group at the time the work was carried out. SHP has received funding from Reckitt, Health Education England/NHS England, West Yorkshire Integrated Care Board, Leeds Hospitals Charity, NIHR, UK MS Society. She is Chair of the Multiple Sclerosis Society Doctoral Fellowship, Early Career Development Awards & Doctoral Training Centres Panel. She is Yorkshire and Humber NIHR Research Delivery Network Regional co-lead for Oral and Dental Health Speciality. N.P. is completing a National Institute for Health and Care Research (NIHR) funded Doctoral Fellowship. He is a member of the ECMID Guideline Committee for penicillin allergy de-labelling. He is a member of the study management committee and is co-ordinator of UK participating sites for the International Network of Antibiotic Allergy Nations (INAAN). L.S. holds fellowship development funding from the Leeds NIHR BRC, was the Chair of the BSACI Penicillin Allergy Working Party, and Co-Lead of the SPACE study. She is Chair of the Perioperative Allergy Network and represents this network on the National Allergy Strategy Group. S.S. is current chair of the British Society for Immunology—Clinical Immunology Professional Network. M.T.K. was a member of the Data Management Committee for the ALABAMA research programme. He was co-author of the British Society for Allergy and Clinical Immunology (BSACI) guidelines on penicillin allergy. He has received research funding from the National Institute for Health and Care Research (NIHR), FSA UK, MRC CiC (via University of Birmingham) and GCRF (via University of Birmingham). J.A.T.S. is a member of the British Society for Allergy and Clinical Immunology Penicillin Allergy working party. He is panel member of the European Society for Clinical Microbiology and Infectious Diseases guideline for antibiotic allergy. He was co-lead for the BSAC massive open access online (MOOC) penicillin allergy course. He has research funding from the Engineering and Physical Sciences Council, NIHR, Wellcome Trust and Medical Research Council. No author directly owns any stocks or shares in a company that might be financially affected by the conclusions of this article. The funder of the research studies associated with this article has not played any decision-making role in the design, execution, analysis or reporting of the research. No assistance from a professional medical writer or similar service has been received. No reimbursement has been accepted by the authors for preparing this article.

## Supplementary data

Supplemental Materials Appendix X available as Supplementary data at *JAC-AMR* Online.
