# Supplementary material for: Widening access to penicillin allergy assessment in the United Kingdom—a proposed implementation plan for the National Health Service (NHS)
Source: JAC Antimicrob Resist. 2026 Jan 7;8(1):dlaf240. doi: 10.1093/jacamr/dlaf240 (PMC12776011; doi:10.1093/jacamr/dlaf240)
Supplement: dlaf240_Supplementary_Data [file dlaf240_supplementary_data.docx]

**Supplemental material from a stakeholder event titled “Widening access to penicillin allergy assessment in the United Kingdom – a proposed implementation plan for the National Health Service (NHS)”**

**Methods**

A core team

Members of both the SPACE and ALABAMA research teams discussed the main messages we wanted to communicate from our work, the lessons and insights that we wanted to share. We considered the level of detail we wanted to convey which was influenced by the range of expertise of those attending the stakeholder event which included members of the general public as well as experts in allergy and antibiotic prescribing as well as non-experts.

SPACE and ALABAMA stakeholder event, London, 27 September 2024

We planned a face-to-face event, in full knowledge that this was the beginning of a process. A diverse group of over 90 attendees was drawn from organisations and professions across the health system and beyond including:

- NHS England *(a publicly funded body that leads the National Health Service in England)*
- UK Health Security Agency *(An executive agency, sponsored by the Department of Health and Social Care which protects public health by preventing, preparing for, and responding to infectious diseases and environmental hazards across the UK)*
- NICE *(Executive non-departmental public body of the Department of Health and Social Care which provides a range of clinical guidance to the NHS in England and Wales)*
- Integrated Care Boards *(NHS organisations responsible for planning and commissioning health services for their local population)*
- NHS Acute Trusts *(organisations that manage hospitals providing urgent and specialist medical care)*
- Electronic health record system providers
- Health charities
- Clinical academics and health professionals from immunology, allergy, anaesthesia, infectious diseases, acute medicine, respiratory, microbiology, pharmacy
- Public and patient involvement in research contributors

Thematic analysis of data

Attendees at the stakeholder event were asked to give written responses to two questions following small table discussions and individual reflection.

Q1. Reflecting on the current situation with regards to the provision of de-labelling for penicillin allergy in the UK, what does success look like in two years’ time? (i.e. what would need to be in place)

Q2. What are the key actions that need to take place to ensure that these markers of success can be achieved? If only three of these actions were possible please identify your top three.

Thematic analysis is a method used to identify, analyse and report on patterns within qualitative data.^[[1]](#endnote-1)^ Template analysis is a flexible form of thematic analysis which emphasises the use of hierarchical coding of data to create a ‘coding template’ which summarises the key features of the data.^[[2]](#endnote-2)^

The following collaborative steps were used to organise and analyse the data collected at the event:

- Attendees’ responses were collated and transcribed into Excel format by the discussion facilitator. (Debbie Cockayne, Page Medical Communications)
- Porter (CEP) and Roleston (CR) coded the responses provided to questions 1 and 2 respectively. This part of the process involved several iterations of coding and discussion.
- From these codes, themes were identified for each question. CEP, CR, Fielding (JF) and Sandoe (JATS) met at regular intervals to assess the preliminary themes, modifying where appropriate.
- CEP and CR then developed a structured, hierarchical ‘summary of data themes’ for their respective questions. They subsequently met with JF to identify any overlap.
- The hierarchical data for both questions was distilled further to create the list of proposed actions as part of the proposed implementation plan. These draft actions were discussed at two subsequent meetings in March 2025, involving members of the ALABAMA and SPACE research teams who organised the London stakeholder event as well as other allergy and electronic health record experts. Where necessary, the actions were refined during and following these research team meetings, before a majority consensus was reached on their inclusion in the proposed implementation plan.
- The agreed actions are summarised in the main paper (Table 1) and outlined in further detail below.
- The full list of hierarchical key data themes is available from the corresponding author upon request.

**Results**

Implementation plan - summary document of actions for widening access to penicillin allergy assessment

| **Actions** | **Proposed responsibility for action** |
| --- | --- |
| 1. **TRANSLATING ASPIRATION INTO ACTION** | |
| Set up and coordinate Penicillin Allergy Network. | Antimicrobial Stewardship and Pharmacy Project Manager (NHS England) |
| Establish Penicillin Allergy Network working groups to deliver on actions in this proposal. | Antimicrobial Stewardship and Pharmacy Project Manager (NHS England) |
| Submit advisory paper to the Department of Health and Social Care Antimicrobial Prescribing, Resistance and Healthcare Associated Infection Group (APRHAI). | Penicillin Allergy Network |
| Work with [National Allergy Strategy group](http://www.nasguk.org/about-us/) to include penicillin allergy in strategic planning. | Penicillin Allergy Network/ National Allergy Strategy group |
| 1. **NATIONAL LEVEL SUPPORT / ENDORSEMENT:** | |
| Request update of National Institute for Health and Care Excellence (NICE) guideline: “Drug allergy: Diagnosis and management of drug allergy in adults, children and young people CG183” to reflect new evidence. | NICE via application from Penicillin Allergy Network |
| Request development of standardised toolkits/resources for non-specialist penicillin allergy assessment. | NICE via application from Penicillin Allergy Network |
| Obtain endorsement for non-specialist delivered penicillin allergy assessment from senior medical authorities across devolved nations (e.g. NHS Chief Medical Officers). | Penicillin Allergy Network |
| Write advisory paper for Department of Health and Social Care Antimicrobial Prescribing, Resistance and Healthcare Associated Infection Group (APRHAI). | Penicillin Allergy Network |
| Ensure collaboration with existing stakeholder groups (e.g. National Allergy Strategy Group, allergy working group and British Society for Allergy and Clinical Immunology, and British Society for Immunology-Clinical immunology professional network, British Society for Antimicrobial Chemotherapy and British Infection Association). | Penicillin Allergy Network |
| 1. **ELECTRONIC HEALTH RECORD(S):** | |
| Develop information technology policy / strategy document for penicillin allergy. | Penicillin Allergy Network/ NHS England/Department of Health and Social Care |
| Work with providers to update electronic health record systems to facilitate documentation of penicillin allergy assessment and align with NICE guidelines. | Electronic health record providers |
| Develop electronic health record-supported patient identification processes. | Electronic health record providers |
| Standardised set of SNOMED codes for penicillin allergy assessment, testing and de-labelling to be added to UK directory. | NHS England Digital |
| 1. **EDUCATION & TRAINING:** |  |
| Provide standardised penicillin allergy assessment training resources for healthcare professionals. | NHS England (Workforce Training & Education Directorate) |
| Identify exemplar case studies of local non-specialist delivery of penicillin allergy assessment. | Antimicrobial Stewardship and Pharmacy Project Manager (NHS England) |
| Develop standardised training model for non-specialist de-labelling workforce e.g. pharmacists. | NHS England (Health Education England) |
| 1. **WORKFORCE & RESOURCES:** |  |
| Ensure all primary care patient records have up-to-date drug allergy histories in line with NICE CG183 standards. | NHS Chief Pharmacist/Integrated Care Boards (NHS organisations responsible for planning and commissioning health services for their local population)/NHS England regional teams |
| Identify local penicillin allergy de-labelling champions. | Antimicrobial Stewardship and Pharmacy Project Manager (NHS England) |
| Establish a record of penicillin allergy assessment services provided by early adopters in the NHS. | Antimicrobial Stewardship and Pharmacy Project Manager (NHS England) |
| Provide local facilities for penicillin allergy assessment and de-labelling. | Integrated Care boards and Local NHS hospital Trusts |
| 1. **RESEARCH NEEDS:** |  |
| Supply the National Institute for Health and Care Research (NIHR) with a paper on research gaps and propose ‘themed funding call’. | Penicillin Allergy Network (research working groups) |
| Specific research gaps identified/ example research questions included:   - which patients or patient groups would benefit most from penicillin allergy assessment - whether patients can be risk stratified according to routine health record data - which patients require specialist allergy- immunology input - burden of penicillin allergy in different ethnic minority groups and development of culturally tailored approaches to enhance patient and community engagement - optimal drug provocation testing dose and duration. | Penicillin Allergy Network (research working groups) |
| 1. **COMMUNICATION PLAN:** |  |
| Public information campaign to raise awareness of the existence of incorrect penicillin allergy labels and their consequences, and what can be done about it. Designed and delivered to minimise unintended consequences. | UK Health Security Agency /Fleming Initiative/Penicillin Allergy Network |
| Communication packs for healthcare professionals and commissioners (Integrated Care Boards) for dissemination to GPs. | NHS England/National Specialty Advisor for Specialised Allergy and Immunology Services |
| 1. **LOCAL IMPLEMENTATION/DELIVERY:** |  |
| Deliver implementation of requirement for up-to-date drug allergy histories in line with NICE CG183 as part of Medicines Optimisation processes (as part of prescribing incentive scheme examples for Integrated Care Boards). | Primary care Medicines Optimisation teams |
| Update National Medicines Reconciliation guidance for hospitals, to include updating antibiotic allergy histories. | NHS England (Chief Pharmacists’ office, Antimicrobial prescribing Medicines Optimisation teams) |
| Develop local communication plan (e.g. present updates at Integrated Care Board’s Pharmacy Leaders webinars). | NHS England (Chief Pharmacists’ office, Antimicrobial prescribing medicines optimisation team) |
| Develop and implement a robust penicillin allergy assessment patient pathway. | NHS England |
| Describe existing models of delivery by early adopters. | Antimicrobial Stewardship and Pharmacy Project Manager (NHS England) |
| 1. **MEASURING SUCCESS (dependant on SNOMED Codes)** | |
| Mechanisms for monitoring performance. | NHS England |
| **PERFORMANCE MEASURES:**   - Number of patients assessed - Number of patients de-labelled - Adverse events associated with assessment recorded performance and safety established - Characteristics of people assessed and de-labelled (incl. ethnicity and co-morbidities) - Risk categorisation of patients assessed and de-labelled documented - Patient acceptability assessed. | NHS England/Integrated Care Boards |

**References**

1. Braun, V., & Clarke, V. (2006). Using thematic analysis in psychology. *Qualitative Research in Psychology,* *3*(2), 77–101. https://doi.org/10.1191/1478088706qp063oa [↑](#endnote-ref-1)
2. Brooks, J., McCluskey, S., Turley, E. *et al* (2015). The Utility of Template Analysis in Qualitative Psychology Research. *Qualitative Research in Psychology,* *12*(2), 202–222. https://doi.org/10.1080/14780887.2014.955224 [↑](#endnote-ref-2)
